# The sympathetic nervous system in the pathophysiology of hypertension: Mechanistic insights and therapeutic implications

**DOI:** 10.1038/s41440-026-02589-6

**Published:** 2026-02-19

**Authors:** Naoyoshi Sakitani

**Affiliations:** 1 Integrated Research Center for Self-Care Technology, National Institute of Advanced Industrial Science and Technology (AIST), 2217-14 Hayashicho, Takamatsu, Kagawa 761-0395 Japan; 2 Health and Medical Research Institute, National Institute of Advanced Industrial Science and Technology (AIST), 2217-14 Hayashi-cho, Takamatsu, Kagawa 761-0395 Japan

**Keywords:** Hypertension, Sympathetic nervous system, Brain, Kidney, Non-pharmacological therapeutic strategy

## Abstract

The sympathetic nervous system plays a pivotal role in the pathophysiology of hypertension. Although the contribution of the sympathetic nervous system to an elevation of blood pressure is well established, the determinants of persistent sympathetic overactivity remain incompletely understood. This review summarizes the findings of recent basic research that have expanded the understanding of sympathetic regulation in hypertension, highlighting emerging mechanisms and providing insights into the development of novel therapeutic strategies.

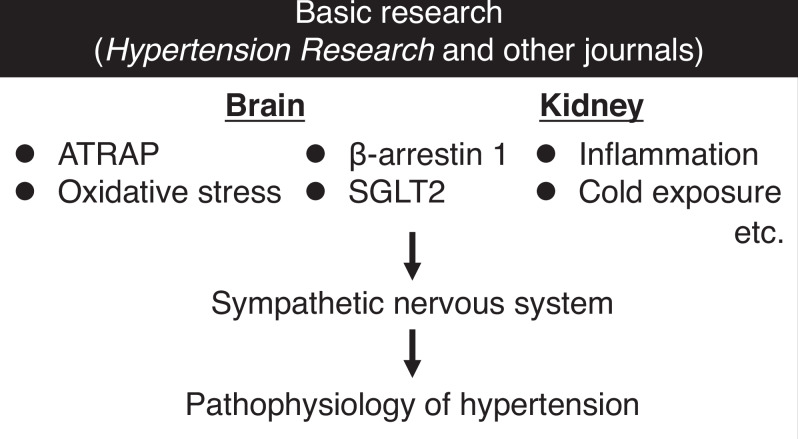

## Introduction

Hypertension is a major global health concern and the leading risk factor for cardiovascular diseases, such as stroke and heart failure. The etiology of hypertension is multifactorial, involving genetic and environmental factors that disrupt the homeostatic systems regulating blood pressure (BP). Although renal sodium excretion systems are recognized major factors involved in the long-term regulation of BP [1], increased activity of the sympathetic nervous system also greatly contributes to the development and progression of hypertension [2].

Sympathetic overactivity elevates cardiac output by increasing heart rate and contractility and induces peripheral vasoconstriction, thereby raising total peripheral resistance [3]. In addition, enhanced sympathetic outflow to the kidneys stimulates renin release and promotes sodium retention [3]. Given these effects, modulation of sympathetic activity is an essential strategy for treating hypertension. Accordingly, autonomic modulation interventions that broadly inhibit sympathetic nerve activity, such as renal denervation, have emerged as potential antihypertensive therapies. However, the variable efficacy of renal denervation reported across clinical studies in certain patient cohorts [4–6] indicates the need for a more detailed understanding of the sympathetic regulation of BP.

This review summarizes recent advances regarding the role of the sympathetic nervous system in the pathogenesis of hypertension, with a particular focus on recently published studies in *Hypertension Research* and other journals. This review also provides discussion on future research directions.

## Regulation of sympathetic nervous activity in the central nervous system

The central nervous system exerts critical control over sympathetic outflow via a network of neural nuclei. This network includes the subfornical organ, the organum vasculosum of the lamina terminalis, the paraventricular nucleus (PVN), the rostral ventrolateral medulla (RVLM), and the nucleus tractus solitarius [7–9]. Among these regions, the PVN and RVLM are particularly important because they serve as major integrative centers linking central sympathetic activity to peripheral sympathetic output. The following sections summarize recent advances in understanding the role of the PVN and RVLM in the sympathetic regulation of BP.

## Role of the paraventricular nucleus in the pathogenesis of hypertension

Angiotensin II (Ang II) type 1 receptor (AT1R) expression in the PVN has been functionally validated, as shown by microinjection of Ang II into the PVN, which induces an increase in renal sympathetic nerve activity (RSNA) through AT1R-mediated signaling [10]. AT1R-associated protein (ATRAP) is an endogenous negative regulator of the AT1R and it promotes AT1R internalization, thereby attenuating Ang II-mediated signaling [11]. Sotozawa et al. showed that enhancing ATRAP expression specifically in the PVN of rats suppressed Ang II-induced hypertension and associated cardiac hypertrophy, suggesting that this effect is mediated by reduced AT1R expression and a consequent decrease in sympathetic activity [12]. Moreover, the importance of ATRAP appears to extend beyond the central nervous system. A recent investigation on the role of ATRAP in the skin showed that the absence of ATRAP in keratinocytes leads to local renin–angiotensin system activation and exacerbated Ang II-dependent hypertension [13]. ATRAP functionally suppresses pathological AT1R overactivation while preserving physiologically beneficial signaling. Therefore, targeting ATRAP could lead to the development of novel therapies that offer a more selective approach than conventional Ang II receptor blockers.

The importance of the PVN in the pathophysiology of hypertension is further highlighted by its role in the developmental programming of hypertension. A study by Hao et al. showed that prenatal lipopolysaccharide exposure increases AT1R expression in the PVN, enhances RSNA, and elevates BP in adult rat offspring [14]. Similarly, Yang et al. reported that gestational diabetes induced hypertension in adult offspring via sympathetic overactivity, which was driven by upregulated AT1R expression in the PVN, as its blockade with losartan attenuated both sympathetic activity and BP [15]. These findings suggest that AT1R signaling within the PVN is a critical factor for translating adverse prenatal conditions into adult hypertension, making it an important therapeutic target for hypertension. In contrast to such detrimental programming, a study by Shan et al. showed the protective effects of exercise as a therapeutic intervention for hypertension. They found that maternal exercise lowered BP in hypertensive offspring by reducing fibroblast growth factor 21 expression, which prevented the detrimental switch of vascular smooth muscle cells to a synthetic phenotype [16]. This finding is particularly important because fibroblast growth factor 21 has been reported to cross the blood–brain barrier [17] and activate the sympathetic nervous system [18]. These findings suggest that maternal exercise involves central sympathoinhibitory mechanisms.

In summary, although the PVN has long been recognized as a critical regulator of sympathetic activity in hypertension, recent studies have advanced understanding of the molecular and developmental mechanisms through which the PVN contributes to regulating BP.

## Role of the rostral ventrolateral medulla in the pathogenesis of hypertension

Oxidative stress in the RVLM contributes to hypertension by increasing sympathetic outflow to peripheral tissues such as blood vessels. A study by Fan et al. showed that Ang II activated the JAK2/STAT3–COX2–PGE2 signaling cascade, which induced local oxidative stress and increased sympathetic activity [19]. This finding suggests that downstream components of Ang II signaling, such as COX2, are potential therapeutic targets for treating hypertension driven by sympathetic overactivity.

In addition to this signaling cascade, the AT1R signaling pathway is regulated by proteins such as β-arrestins. β-arrestins negatively regulate G protein-coupled receptors by promoting desensitization and endocytosis and by mediating distinct intracellular signaling pathways [20]. The importance of these proteins in hypertension is highlighted by evidence that the overexpression of β-arrestin 1 in the RVLM reduces BP and sympathetic outflow in spontaneously hypertensive rats, and this effect is associated with downregulation of AT1R [21]. This sympathoinhibitory role of β-arrestin 1 is also evident in postmenopausal hypertension. Yan et al. reported that the downregulation of β-arrestin 1 in the RVLM of ovariectomized rats led to sympathetic overactivation and elevated BP [22]. These findings suggest that β-arrestin 1 is a potential therapeutic target in hypertension. Recent research has provided further insights by identifying upstream mechanisms regulating β-arrestin 1. A study by Wang et al. identified miR-22-3p, potentially released from microglia, as a critical negative regulator of β-arrestin 1 in the RVLM of hypertensive rats [23]. The involvement of microglia in the AT1R signaling highlights neuroinflammation as another critical factor in sympathetic regulation within the RVLM. Furthermore, Yin et al. showed that microbiota-derived acetate lowered BP by inhibiting neuroinflammation and sympathetic activity through its regulatory effects on microglia and astrocytes in the RVLM of spontaneously hypertensive rats [24]. These studies strongly suggest that regulating microglia-mediated neuroinflammation in the RVLM is a crucial therapeutic target for hypertension.

Treatment with sodium-glucose cotransporter 2 (SGLT2) inhibitors has been shown to reduce both blood glucose concentrations and BP in hypertensive patients with diabetes. Oshima et al. investigated a potential novel mechanism underlying these antihypertensive effects and showed that SGLT2 inhibitors suppress the activity of RVLM neurons in experiment models [25]. This finding suggests that, in addition to promoting renal glucose and sodium excretion, SGLT2 inhibitors may modulate central sympathetic outflow. Additionally, another class of diabetes medications, glucagon-like peptide-1 (GLP-1) agonists, have been investigated to determine their effect on the sympathetic nervous system. Xu et al. investigated the role of GLP-1 and GLP-1 receptor within the PVN in rats and found that activation of GLP-1 receptor in the PVN led to an increase in sympathetic activity and BP [26]. Although this study focused on the PVN, it suggests a role for GLP-1 signaling in central sympathetic control. Future studies are warranted to investigate whether a similar mechanism exists in other key nuclei, including the RVLM. However, these central mechanisms have been shown primarily in animal and ex vivo experimental models, and their relevance in humans has not yet been clearly established.

Overall, recent investigations have reinforced the established importance of the RVLM in sympathetic regulation while revealing additional molecular and cellular mechanisms implicated in hypertension.

## Regulation of sympathetic nervous activity in the kidney

While the central nervous system plays a critical role in sympathetic regulation, accumulating evidence indicates that the kidney is not merely a target but also a source of sympathetic activation. This section summarizes recent findings on diverse factors that modulate RSNA and contribute to the development of hypertension.

A study by Baumann et al. showed that the antihypertensive effect of renal denervation was primarily mediated by afferent renal nerves in a deoxycorticosterone acetate-salt hypertensive mouse model [27]. They also found that blocking inflammatory signaling through the interleukin-1 receptor (IL-1R), either genetically or pharmacologically, produced a BP-lowering effect equivalent to that of afferent renal denervation [27]. Notably, combining both interventions produced no additive effect, confirming that IL-1R signaling is a key mechanism within the afferent renal nerve pathway that drives deoxycorticosterone acetate–salt hypertension [27]. These findings emphasize the contribution of renal inflammation to afferent nerve activation and highlight inflammatory signaling as a promising therapeutic target.

While it is well established that cold exposure increases BP via sympathetic activation, the underlying mechanisms have not been fully elucidated. Recent research by Yoshimoto et al. provided critical insights into the specific roles of the renal sympathetic nerves in cold-induced hypertension [28]. They found that four days of cold exposure (10 °C) triggered distinct sympathetic responses. However lumbar sympathetic nerve activity, arterial pressure, and heart rate increased only transiently, and RSNA showed a progressive rise throughout the cold period [28]. Importantly, this heightened RSNA persisted even after the rats were returned to a warmer environment (24 °C) [28]. These results suggest that increased RSNA is a critical driver of cold-induced hypertension and provide a physiological explanation for seasonal variations in BP and the higher cardiovascular risk observed under cold environmental conditions.

Clonal hematopoiesis (CH), which is an age-related condition characterized by the expansion of hematopoietic stem cells with somatic mutations in the absence of overt hematologic malignancy, is a well-known risk factor for multiple pathologies. These pathologies include cardiovascular diseases, such as myocardial infarction and stroke, autoimmune conditions, and osteoporosis. Recently, evidence suggesting a relationship between CH and hypertension has been reported. Polizio et al. investigated the association between CH and hypertension, and identified a mechanism involving the sympathetic nervous system [29]. They found that TET2-CH promotes hypertension through an NLRP3 inflammasome-mediated increase in RSNA [29]. On the basis of these results, they suggested that screening for CH driver mutations, such as TET2, could serve as a useful biomarker strategy to identify patients who would preferentially benefit from catheter-based renal nerve ablation [29]. This finding is clinically important because it may help overcome a major challenge in identifying suitable candidates for renal denervation, which is currently limited by the inability to directly measure renal sympathetic activity.

Taken together, these studies indicate that the kidney serves not only as a target but also as a source of sympathetic activation, with inflammatory, environmental, and genetic factors contributing to sympathetic overactivity and the pathogenesis of hypertension.

## Emerging non-pharmacological therapeutic strategies

Despite the development and proven efficacy of various antihypertensive medications, optimal BP control is not consistently achieved in clinical practice. Similar to other pharmacological agents, antihypertensive drugs have potential side effects and contraindications. In addition to pursuing new molecular targets, recent studies have also investigated alternative strategies to modulate sympathetic nervous activity. In particular, there has been growing interest in non-pharmacological interventions as promising approaches for the safe and effective management of BP.

It is well established that physical exercise exerts an antihypertensive effect. However, the mechanisms through which exercise exerts a positive effect on BP control remain unclear. A recent study by Murase et al. showed that mechanical stress, specifically vertical acceleration generated during fast walking or slow jogging, induced interstitial fluid flow in the brainstem and exerted shearing forces on cells in the RVLM, thereby reducing AT1R expression in astrocytes and lowering sympathetic activity and BP in hypertensive rats [30]. Furthermore, hypertensive adults who underwent vertically oscillating chair riding, mimicking the vertical acceleration experienced during light jogging, also exhibited BP reduction [30]. These findings indicate that mechanical interventions may represent a novel strategy for BP reduction through the suppression of sympathetic activity.

## Conclusion and future perspectives

In conclusion, the sympathetic nervous system is a central driver of hypertension, and recent advances have improved the understanding of its regulation within the PVN, RVLM, and kidney. A variety of factors converge on these regions and contribute to sympathetic overactivity. These insights could lead to novel molecular targets and highlight the potential of non-pharmacological approaches, such as exercise and mechanical interventions, as complementary strategies. Taken together, these findings provide a mechanistic foundation for the development of more effective and personalized approaches to BP control.
